# Neurodiversity: A Behavior Analyst’s Perspective

**DOI:** 10.1007/s40614-025-00435-7

**Published:** 2025-02-18

**Authors:** Michael Nicolosi, Karola Dillenburger

**Affiliations:** 1 Data Driven ABA, Schaffhausen, Switzerland; 2 Centre for Behaviour Analysis, Queen’s University Belfast, Belfast, UK

**Keywords:** Autism, Neurodiversity, Neurodiversity movement, Neurodivergence, Applied behavior analysis

## Abstract

A neurodiversity movement (NDM) has gained momentum, mainly driven by autistic self-advocates. The main argument of the NDM is that neurodivergent people experience discrimination that is on par with the historical discrimination of other minority groups. In this article, we propose a behavior analyst’s perspective on the NDM. We first explore the history and emergence of the concept of neurodiversity and its neurological as well as psychological basis. We consider its potential for generating what some consider a zero-sum game, in which one group makes all the gains potentially at the expense of another group. We finish with the suggestion that a win–win situation is possible if the focus shifts proactively to advocacy for all persons with autism, including those with very high support needs who often are not able to advocate actively for themselves and who tend to benefit greatly from evidence-based behavior-analytic interventions.

The saddest aspect of life right now is that science gathers knowledge faster than society gathers wisdom. Isaac Asimov (Asimov & Shulman, 1988).

The universal diagnostic label “autism spectrum disorder” (ASD) was introduced in 2013 in the fifth edition of the *Diagnostic and Statistical Manual of Mental Disorders* (*DSM-V*; American Psychiatric Association, 2013). The *DSM-V* amalgamated various previous diagnoses, including Asperger’s syndrome (Asperger, 1944) and childhood autism (Kanner, 1943) described in the *DSM-IV* under “pervasive developmental disorders.” With only one diagnostic category available, the ASD diagnosis now includes individuals with autism who have relatively well-developed skills and who essentially are able to advocate for themselves and navigate the world more or less unaided. These highly articulate adult autistic self-advocates tend to be the drivers of the neurodiversity movement (NDM). The ASD diagnosis now also includes vulnerable, profoundly affected individuals (Lord et al., 2022), who frequently do not have the most basic functional social or life skills and usually cannot live independently. The latter group tends to live with their aging parents or in residential care (Dillenburger & McKerr, 2009). The difference in terms of support needs between these groups is significant. Thus, an assessment of the level of support needs is part of the medical diagnosis in the *DSM-V*.

Despite the shift in diagnostic classification, it is important to remember that the diagnostic label of ASD relies entirely on observations of behavior. In a typical situation, the diagnosis is confirmed after detailed assessments of developmental history, clinical observations of behavior, caregiver or self-reports, and/or formal testing, that may include ASD-specific screening (e.g., Childhood Autism Rating Scale [CARS], Schopler et al., 1980; Autism Diagnostic Interview-Revised [ADI-R], Lord et al., 1994). However, self-identification without clinical diagnosis is becoming increasingly prevalent among adult self-advocates (Sarrett, 2016). It is intriguing that this differentiation is ignored by some (cf, Dillenburger, 2025).

## The Emergence of the Neurodiversity Movement

The Australian sociologist Judy Singer is credited with coining the term “neurological diversity” in 1998 to describe people with various diagnoses, including autism (specifically Aspergers syndrome), attention deficit hyperactivity disorder (ADHD), and dyslexia (Singer, 1998, 2017). At the time, her writing only received limited attention because her “personal exploration” was submitted as part of her undergraduate degree; as with most bachelor’s theses, it did not receive much publicity (Harris, 2023).

Nevertheless, some highly articulate autistic adults picked up on Singer’s idea of a “new social movement based on neurological diversity” and started to produce social media posts and papers about the way autistic people were being discriminated against. The “neurodiversity movement” (NDM) began to thrive, particularly in the early 2020s, when COVID-19 lockdowns invigorated the kinds of virtual interactions that frequently are a preferred method of communication (Harris, 2023). This provided ideal platforms, via social media, blogs, Twitter and Facebook feeds, message boards, and the rapid dissemination of open access papers, for the NDM to grow (Singer et al., 2022).

NDM campaigners argued that autism was not a disability as classified in the *DSM-V*, but primarily a neurological difference. They identify as neurodivergent or “differently wired” and at times, state that they are “proud to be autistic.” They called for an “autistic culture” to develop, similar to that of the deaf community, aiming to achieve full inclusion of neurodiverse individuals in society, via societal respect, acceptance, and environmental adaptations (Hughes, 2016). The NDM agrees that autistic people should be unified under one label^1^ and argue that there should be no distinction between levels of functioning or support needs (Vance, 2022). In line with the concept of “difference” rather than “pathology,” the NDM supports a notion that certain diagnoses including autism, attention deficit hyperactivity disorder (ADHD), dyslexia, anxiety, obsessive–compulsive disorder, depression, intellectual disability, schizophrenia (Hughes, 2016) are best thought of as normal variations of human existence (Russell, 2020) and, as such, they require acceptance rather than therapy or intervention.

Some of the core features of the NDM are well expressed in the following passage from Sinclair (1993) in an early essay titled “Don’t Mourn for Us”:

Autism isn't something a person has, or a "shell" that a person is trapped inside. There's no normal child hidden behind the autism. Autism is a way of being. It is pervasive; it colors every experience, every sensation, perception, thought, emotion, and encounter, every aspect of existence. It is not possible to separate the autism from the person—and if it were possible, the person you'd have left would not be the same person you started with. (p. 1)

Thirty years on, his passage still is considered “a touchstone for the neurodiversity movement” (Kapp, 2020, p. 23). It views autism firmly as part of the biological make-up of the person, yet the NDM generally rejects the medical model. The medical model focuses on organismic factors that lie within the individual, whereas at the core of the social model is the principle that people are disabled not because of their physical impairments, but because of barriers imposed by society (Bunbury, 2019). The general shift away from the medical model towards the social model (United Nations, 2006) has reduced societal barriers, changed the media image of people with disabilities, achieving changes to the legal system, and even introduced universal design in public buildings (e.g., ramps, large print leaflets) and transport (Cree & Davis, 2021).

There is a contradiction, however. Although proponents of the NDM commonly expresses a preference for the social model, their emphasis is on organismic neurological differences that lie within the individual clearly emanates from the medical model. When autism is viewed as an integral part of the person’s and neurological make-up, for example, when talking about the “autistic brain” (Russell, 2020; Silverman, 2011), the focus is on the medical model. As Koi (2021) pointed out: “The relationship of autistic and ADHD people and their families with genetics research is complex and largely shaped by the connection of genetic research with the medical aim of ‘curing’ individuals of their autism or their ADHD” (p. 55).

No biomarkers, either genetic or neurological, have yet been discovered to distinguish between people with autism and those without autism (Hodges et al., 2020). Moreover, the neurology of every individual person differs and there is no neurological “normality” (Masini et al., 2020). As such, neurological diversity is characteristic of all humans, a fact the term “neurodivergence” was coined to account for (Legault et al., 2021), though is perhaps better captured by the term “human diversity” (Ekblad, 2013).

## Controversies and Debates

The NDM objects to all types of social barriers, exclusion, oppression, and stigmatization (Kapp, 2020), especially regarding social rules developed by nonautistic, neurotypical people that, according to the NDM, are not sensitive enough to the needs of neurodivergent people (Woods, 2017). Any insistence that everyone should behave in accord with neurotypical social rules is termed “ableism” (Kapp, 2020). The idea that being “normal” or neurotypical is better than being any other way is termed “normalization” (Milton, 2012). It is argued that ableism and normalization lead to discrimination of anyone unable or unwilling to adhere to normative social rules and customs. Rather than everyone adjusting to an ableist normalization agenda, NDM activists call for society to learn to accommodate and value different ways of being human. In the twenty-first century, most reasonable people, neurodivergent or neurotypical (Ekblad, 2013), probably would agree with this position. Societal acceptance, tolerance, accommodation, and inclusion are vitally important, not only for the individual, but for society as a whole (Skinner, 1977, 1999).

It is perplexing, however, that some people in the NDM argue that anyone who tries to teach a new behavior to a person is trying to change the person, thus not accepting the person as they are. Thus, any attempt by parents or professionals to educate a child, to teach them new skills, or to implement interventions that focus on enhancing independence and inclusion or reducing behavioral challenges, are considered as ableist and discriminatory, based on a normalizing agenda (Kapp, 2020). In this context, some NDM activists have set their sights particularly on interventions delivered by behavior analysts (Milton, 2012). This is despite the overwhelming evidence that these interventions can enhance quality of life immensely, and at times can be lifesaving (Larsson, 2021). Indeed, some in the NDM propose that applied behavior-analytic interventions should be modified, or better still, abandoned altogether—what has been termed the abolitionist neurodiversity critique (Graber & Graber, 2023).

Vyse (2022) called out this zero-sum game:

It is one thing to form a social movement in an effort to gain greater acceptance of and better supports for a group of people in need. It is quite another to do so at the expense of another group who is also in great need. Helping people on the autism spectrum should not be a zero-sum game with gains at one end of the spectrum requiring losses at another. Unfortunately, the autism self-advocacy movement’s attacks on ABA [applied behavior analysis] create just such a dilemma. (p. 27)

We agree with Vyse that there is no need for a zero-sum/winner-takes-all mentality. We believe that a win–win position is not only possible, but consistent with the *Ethics Code of Behavior Analysts*, based on four core principles: “benefit others and do no harm; treat others with compassion, dignity, and respect; behave with integrity; and ensure their competence” (Behavior Analyst Certification Board [BACB], 2023, p. 4). In fact, it is coherent with Judy Singer’s own views (Lutz, 2023a).

It is clear, then, behavior analysts have much in common with the NDM by emphasizing the importance of inclusion, compassion, and acceptance. In addition, behavior analysts ensure that their work benefits others and does no harm. Moreover, behavior analysts realize the limitations of the medical perspective (Dillenburger et al., 2013; Skinner, 1977) and expose the language traps that leave people looking for explanations of behavior in the wrong place (Keenan & Dillenburger, 2020a, 2020b; Moore, 2013). Rather than engaging in mentalism (e.g., circular reasoning; Schlinger, 2003; Squier et al., 2010), behavior analysts explain behavior by analyzing the circumstances of which behavior is a function—an approach that may be seen as firmly based in the social model (Bunbury, 2019). Given that the diagnostic category of autism is purely behaviorally based (APA, 2013), behavior analysts view the term “autism” as a summary label (Cooper et al., 2020) for specific behaviors, namely related to repetitive restricted behavior and social communication. A classic turn of phrase often used to communicate this point is “you are your behavior.”

Given that the NDM considers autism as immutable and unmodifiable part of the autistic person’s biology, any attempt at modification of autism-related behavior is deemed detrimental to the person’s integrity. Hence, some in the NDM have rejected any kind of behavior-analytic intervention (Milton, 2012). In contrast, behavior analysts view a person as always evolving and therefore consider learning new behavior as an integral part of being human and lifelong human development. In order to promote socially important behavior (Baer et al., 1968, 1987), inclusion, independence, and quality of life (Baer, 2004; Dillenburger & Coyle, 2019), behavior analysts develop carefully designed, individually tailored, evidence-based interventions. In effect, behavior analysts contend that there are evidence-based interventions that should be made available, if and when needed, to maximize an individual’s quality of life, independence, and freedom (Baer, 2004).

Although the NDM proposes that autism is part of the person and therefore essentially a lifelong condition, behavior analysts have accumulated considerable evidence that some people diagnosed with autism in childhood no longer meet diagnostic criteria as they grew older. This development is strongly correlated with receiving good quality early intensive behavior-analytic interventions that facilitate skills development, especially social interaction and communication (Fein et al., 2013; Orinstein et al., 2014). This is not the same as saying that what they provide is a “cure” for autism, because behavior analysts do not view autism as an illness that requires a cure. Rather, the term autism is a summary label for behaviors that can change, adapt, and grow, given the right social and environmental contingencies and adjustments (Brodhead et al., 2018). In the same vein, in the early days of autism interventions, terms such as “indistinguishable from peers” were used, but this did not mean that children with autism were “normalized” or “cured.” What it meant was that these children had developed life-skills similar to those of all other children of their age, enabling them to make friends, go to school unaided, and take part in other everyday activities that previously had alluded them.

There are numerous peer-reviewed scientific articles describing how behavioral interventions successfully enhance the skills of children and adults with autism (Larsson, 2021; Myers et al., 2007), increase intellectual functioning, social and communication skills, and independence at home, in school, and in the community (Eikeseth et al., 2015; Howard et al., 2014; Nicolosi & Dillenburger, 2022). This body of research shows that people with autism can learn all sorts of skills if their environment is adequately adjusted and supported by family and caring professionals.

## Representation and Advocacy

There are a significant number of parents and caregivers who have pointed out that the NDM does not represent their loved ones with profound autism (deBoer, 2022; Kansen, 2016; Lamb, 2023; Lutz, 2013, 2023a, 2023b; Mitchell, 2019). In fact, in an interview with Judy Singer, Lutz reported that she said so herself:

“I was very clear in my thesis that I was only talking about Asperger’s,” Singer told me. And in fact she couldn’t have been more explicit: In an early section entitled “Notes on language,” she wrote, “I want to make it clear that when I used the term ‘autistic,’ I am referring only to people with what is called High-Functioning Autism (HFA) or Asperger’s Syndrome (AS), that is, people who have normal to high ‘intelligence.’” Singer made this distinction because it was obvious to her that the challenges faced by the “brainy but socially inept nerds” at the center of her thesis were both qualitatively and quantitatively different from the profound impairments that characterized classic autism. “What is now called autism isn’t a unitary condition, and I only know Asperger’s—I can’t speak for severe autism.” (Lutz, 2023a)

The relatively common co-occurrence of intellectual disability, the frequent presence of serious challenging and self-injurious behaviors, the pervasiveness of self-stimulatory behavior, and the relatively low level of basic life skills in persons with profound autism, mean that the majority of these people require lifelong support and assistance. These are the people that usually benefit greatly from behavior-analytic supports and interventions (Fein et al., 2013; Orinstein et al., 2014) and who do not tend to learn skills unless explicitly taught (Howlin et al., 2004, 2014). Given the relative scarcity of behavior analysts worldwide, families of individuals with profound autism often find it difficult to locate good quality providers of behavior-analytic services (Kelly et al., 2019); and parents often have to fight the system to try and ensure that their children can benefit from behavior-analytic interventions (Blakemore, 2021; Byrne & Byrne, 2000).

It is unlikely that highly able autistic self-advocates would require intensive behavioral intervention services, even if they were available. Yet, some in the NDM have claimed that they have personal experience with poorly practiced behavior-analytic services (Leaf et al., 2018). Although behavior analysts are generally highly skilled and adhere to a very strict ethical code, due to lack of professional regulation worldwide, the possibility of poor practice must be acknowledged. The U.S.-based Behavior Analyst Certification Board (BACB, 2018) oversees board certified behavior analysts (BCBA) in the United States and Canada and therefore their disciplinary procedures apply in cases of malpractice that break the BACB *Ethics Code*. Similar regulations exist in the UK (Professional Standards Authority, 2023). However, few other countries have a legal framework for professional regulation of behavior analysts (Keenan et al., 2023; Kelly et al., 2019) and Thus the profession remains largely unregulated internationally (Kelly & Trifyllis, 2022). Lack of regulation, together with limited training opportunities and widespread misinformation about behavior analysis, can lead to a perfect storm, where the whole science of applied behavior analysis is dismissed (Baron-Cohen, 2014; Milton, 2012; The Skeptical Advisor, 2014).

## Suggested Solutions

We propose two possible solutions. First, the term “neurodivergent” could be employed for those who self-identify under this term and who do not have a clinical diagnosis and do not require clinical support services. Those who identify as neurodivergent would be able to advocate for societal changes that would lead to much better inclusion and acceptance of the diversity of the human spectrum (Ekblad, 2013). This change would free the clinical diagnosis of autism or ASD as outlined in the *DSM-V* for those who have high clinical support needs and who are professionally diagnosed by qualified clinicians as meeting the relevant diagnostic criteria (APA, 2013). This would enable this latter group to benefit from services provided by those with certified university training in ethically sound, scientifically validated, evidence-based clinical interventions (Applied Behavior Analysis Available for All, 2022).

On the other hand, the NDM could evolve into a movement that represents the entire autistic spectrum, rather than privileging those with the skills to self-identify and self-advocate. This would not only ensure that all people with autism are represented adequately in the NDM, but it would enable full collaboration with all stakeholders, including behavior analysts, involved in the struggle for equal rights of people with autism. It would enable the focus of synergetic energies of the movement to fight the real nemeses: discrimination and societal inertia (cf, Dillenburger & Keenan, 2023).

## Data Availability

Data sharing is not applicable to this article because no new data were created or analyzed in this study.

## Footnotes

1 NB. They prefer the term “autism,” rather than “autism spectrum disorder”.
